# Comparison of interobserver and Intraobserver reliability of alpha angle measurements using different types of circles for femoroacetabular impingement syndrome

**DOI:** 10.1093/jhps/hnaf006

**Published:** 2025-01-30

**Authors:** Özgür Aydin, Onur Hapa, Emre Acar, Mustafa Celtik, Selahattin Agca, Cihangir Turemis, Raif C Yarol, Selahaddin Aydemir

**Affiliations:** Department of Orthopaedic Surgery, Faculty of Medicine, Dokuz Eylul University, İnciralti, Mithatpasa St. No:56 Balcova, Izmir 35330, Turkey; Department of Orthopaedic Surgery, Faculty of Medicine, Dokuz Eylul University, İnciralti, Mithatpasa St. No:56 Balcova, Izmir 35330, Turkey; Department of Orthopaedic Surgery, Faculty of Medicine, Dokuz Eylul University, İnciralti, Mithatpasa St. No:56 Balcova, Izmir 35330, Turkey; Department of Orthopaedic Surgery, Dr. Abdurrahman Yurtaslan Oncology Training and Research Hospital, Demetevler, 354/1. St., Yenimahalle, Ankara 06200, Turkey; Department of Orthopaedic Surgery, Faculty of Medicine, Dokuz Eylul University, İnciralti, Mithatpasa St. No:56 Balcova, Izmir 35330, Turkey; Department of Orthopaedic Surgery, Alper Cizgenakat State Hospital, Boyalık Mevki, Fahrettinpasa, Cesme-ilica Road 2/A, Izmir 35930, Turkey; Department of Radiology, Faculty of Medicine, Dokuz Eylul University, İnciralti, Mithatpasa St. No:56 Balcova, Izmir 35330, Turkey; Department of Orthopaedic Surgery, Faculty of Medicine, Dokuz Eylul University, İnciralti, Mithatpasa St. No:56 Balcova, Izmir 35330, Turkey

## Abstract

The alpha angle is a radiological measurement that has been proposed for the detection of cam morphology in patients suspected of femoroacetabular impingement syndrome (FAIS). After analysing published articles on FAIS, it is apparent that different types of circles are used in the measurement of alpha angles. To determine the interobserver and intraobserver reliability of alpha angle values measured using different types of circles on a 45° Dunn lateral radiograph in patients with symptomatic FAIS. The 45° Dunn lateral radiographs of the operated hips of patients who underwent hip preservation surgery in Dokuz Eylül University Hospital between 2014 and 2017 were evaluated retrospectively. Alpha angles were measured manually with transparent goniometers using three different circle types: thin full circle, thin-dashed circle, and thick full circle, and eye estimation without circle insertion. Measurements were made separately by an orthopaedist and a radiologist. A second measurement was made by the radiologist 1 month later for intraobserver reliability. Interobserver reliability for the thin full circle, thin-dashed circle, thick circle, and eye estimation (ICC = 0.645, 0.525, 0.494, and 0.588, respectively). Intraobserver reliability for the thin, thin-dashed, thick line, and eye estimation (ICC= 0.626, 0.681, 0.681, and 0.462 respectively). Interobserver difference of mean values of alpha angles for thin full, thin-dashed, thick full, and eye estimation were 2.7°, 0.9°, 1.1°, and 2.3°, respectively. Intraobserver differences between measurements were 1.5°, 0.9°, 0°, and 1.6°, respectively. Our study demonstrated that interobserver reliability is highest when measurements are made using a thin full circle.

## Introduction

**Figure 1.**
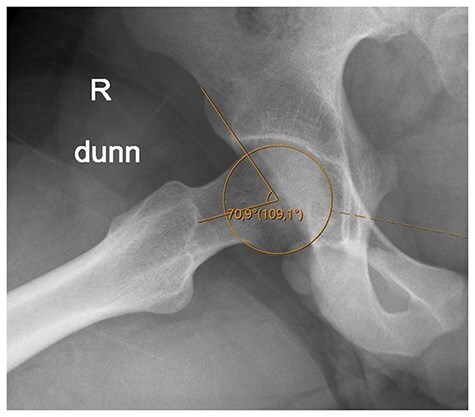
Alpha angle measurement.

FAI syndrome is a motion-related clinical disorder of the hip with a triad of symptoms, clinical signs and imaging findings. It represents symptomatic premature contact between the proximal femur and the acetabulum [1] and can cause osteoarthritis at a young age [2–4]. It arises due to two basic disorders in hip morphology: (i) cam morphology of the femur, caused by loss of the normal concavity of the anterior head–neck junction, and (ii) pincer morphology of the acetabulum due to localized overgrowth of the acetabular margin with overcoverage of the femoral head. These deformities may also occur in combination [3, 4]. The presence of a cam deformity is commonly defined by the location of the junction between the spherical contour of the femoral head and the cortical surface of the femoral neck, as seen on imaging studies. The most popular index for defining the extent of the spherical head surface, and thus the presence and severity of a cam deformity, is the alpha angle, which is defined as the angle between the neck axis and a line joining the spherical head–neck junction and the head centre (Fig. 1). However, several studies in the literature have shown that radiographic measurements of the alpha angle can be highly variable, suggesting a need for methods that increase the reliability of the measurement of the alpha angle [5–7]. Nötzli *et al*. [8], Rakhra *et al*. [9], Sutter *et al*. [10], and Morris *et al*. [11] published studies in which alpha angle measurements were made with thin dashed circles. Smit *et al*. [12], Carlisle *et al*. [13], and Barton *et al*. [14] published studies containing figures in which alpha angle measurements were made with thin full circles. Nepple *et al*. [15] published studies in which alpha angle measurements were made with thick full circles. Therefore, in this study, we aimed to investigate the intraobserver and interobserver reliability of alpha angles measured with different types of circles.

## Materials and methods

**Figure 2.**
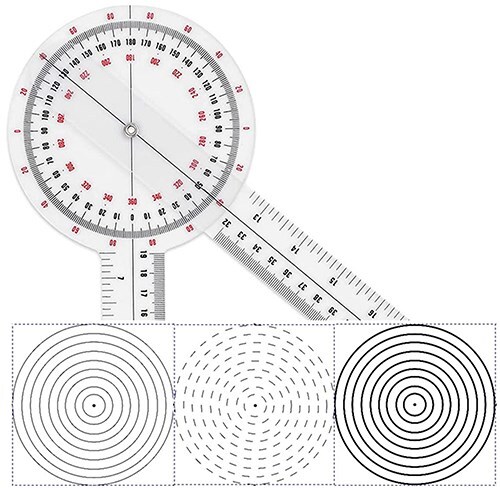
Transparent goniometer and the types of circles that were used in these goniometers.

**Figure 3.**
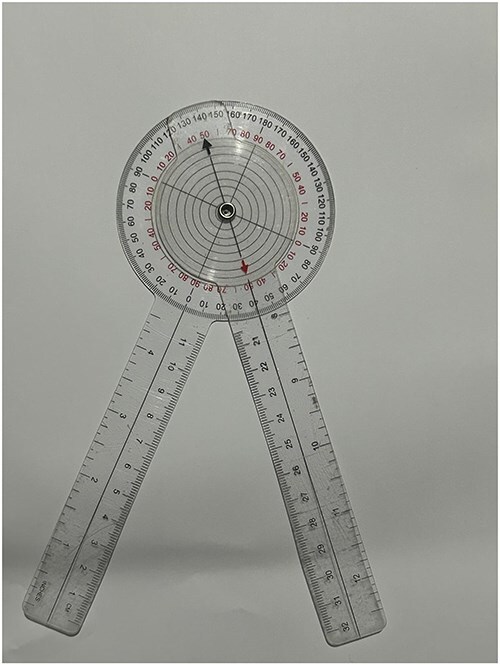
Transparent goniometer with thin-full circles on it.

**Figure 4.**
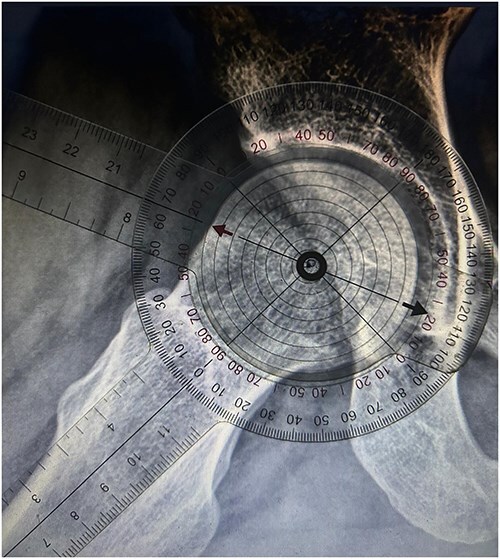
Alpha angle measurement with a thin-full circle.

Ethics committee approval was obtained for this study. Plain radiographs of patients who underwent arthroscopic hip preservation surgery for cam-type FAIS at Dokuz Eylül University Hospital between 2014 and 2017 were retrospectively analysed. 45° Dunn radiographs of 59 patients were evaluated and alpha angle measurements were performed separately by an orthopaedic surgeon and a radiologist. The radiologist repeated the measurements 1 month later to assess intraobserver reliability. The measurements were made on digital images using the hospital’s Picture Archiving and Communication System through SECTRA IDS7 with manual measurement procedures using transparent goniometers with printed circles. To measure the alpha angle, as outlined in Nötzli’s original article [8], a circle that best fits the femoral head was identified, along with its centre and the anterosuperior point protruding beyond the boundary of the circle. Two lines were then drawn: one from the circle’s centre to the protruding point outside the circle, and another from the centre to the midpoint of the narrowest part of the femoral neck. The angle formed by these two lines was subsequently measured. This process was repeated separately for each circle style and each radiograph during the measurement. With this measurement method, in addition to three different types of circles, measurements were also made by eye estimation without inserting circles. The following three types of circles and eye estimation were used. The first type: a classic thin full circle with continuity. The second type: a dashed form of the classic thin circle. The third type: a 5-fold thickened version of the classic thin circle. Eye estimation method: in this measuring method, the point extending from the circle and the centre of the circle was determined by the raters’ estimation without placing a circle (Fig. 2). These circles were placed in the centre of the transparent goniometers (Fig. 3). Three different goniometers were made and used to perform the measurements manually on digital images (Fig. 4). The thickness of the thin ring and the thin dashed was 0.300 points (∼0.1 mm). The thin-dashed circles’ gaps made up 40% of the circumference. The line thickness of the thick ring was 1.5 points (∼0.5 mm). Both raters measured the alpha angles four times using three different goniometers and eye estimation. The radiologist measured the alpha angles of the same radiographs again 1 month later. The assessment of interobserver reliability was the main interest. Intraobserver reliability was performed with the radiologist’s assessment as a secondary outcome.

## Statistics

The data were analysed using the statistical package program IBM SPSS Statistics Standard Concurrent User V 29 (IBM Corp., Armonk, NY, USA). Descriptive statistics are expressed as number of units (*n*), percentage (%), and mean ± standard deviation. The normal distribution of the data of numerical variables was assessed using the Shapiro–Wilk normality test. The homogeneity of variance was analysed using the Levene test. Differences between measurements between and within raters were compared using a two-way analysis of variance on repeated measures. Bonferroni correction was applied for multiple comparisons. The compatibility of the measurements between the raters was evaluated with the two-way mixed model, and the interrater correlation coefficient was calculated with the absolute agreement type. The agreement between the first and second measurements of the raters was evaluated with the intrarater correlation coefficient calculated with the two-way mixed model and consistency type. A value of *P* < .05 was considered statistically significant.

## Results

A total of 33 right hips, 26 left hips of 59 patients, 19 female, 40 female, with a mean age of 35.3 ± 11.1 (range 19–58) were assessed in this study.

Table 1 compares the differences between the raters for each measurement for each method and the differences between the second rater for the first and second measurements.

**Table 1.** Interrater and intrarater comparisons for each method.

		Raters		*P*-values*		
	Measurement	Rater-1	Rater-2	*P* ^&^	*P* ^1^	
Thin full circle	1	70.4°± 6.8°	73.1°± 7.7°	0.009		
	2	-	71,6°± 6,5°	-	<.001	
Thin dashed circle	1	70.2°± 6.8°	71.1°± 7.3°	0.387		
	2	-	70.2°± 6.3°	-	<.001	
Thick full Circle	1	69.2°± 6.8°	68.1°± 6.8°	0.325		
	2	-	68.1°± 5.9°	-	0.877	
Eye estimation	1	69.9°± 6.7°	72.2°± 8.6°	0.048		
	2	-	70.6°± 6.7°	-	0.001	

* : Analyses were performed with a two-way analysis of variance in repeated measurements. Bonferroni correction was applied for multiple comparisons, *P*^&^: interrater comparisons, *P*^1^: comparison of first and second measurements for the second rater.

According to Table 1, the mean values of the alpha angle using the thin full circle were 70.4°and 73.1°. Using the thin-dashed circle, they were 70.2°and 71.1°. Using the thick full circle, they were 69.2°and 68.1°, and using eye estimation they were 69.9°and 72.2° for the first and second measurers, respectively. The alpha angles were 73.1° and 71.6° for thin full circle, 71.1° and 70.2° for thin-dashed circle, 68.1° and 68.1° for thick full circle, and 72.2° and 70.6° for eye estimation for the first and second measurements made by the radiologist, respectively.

Table 2 demonstrates the values of interobserver reliability between the first and second raters and the values of intraobserver reliability for the second rater, depending on the measurement method. The ICC values of interobserver reliability for thin full circle, thin-dashed circle, thick full circle, and eye estimation without circle insertion measurement methods were 0.645, 0.525, 0.494, and 0.588, respectively. While poor agreement was found for the thick circle, moderate agreement was found for the other three methods.

**Table 2.** Interrater and intrarater correlation coefficient with 95% confidence interval.

	Thin circle	Thin-dashed circle	Thick circle	Eye estimation
Interrater^‡^	0.645 (0.404–0.788)	0.525 (0.202–0.718)	0.494 (0.164–0.696)	0.588 (0.308–0.755)
Rater-2^†^	0.626 (0.441–0.759)	0.681 (0.516–0.797)	0.681 (0.516–0.797)	0.462 (0.235–0.641)

‡ : Interrater correlation coefficient.

† : Intrarater correlation coefficient.

ICC values of intraobserver reliability for thin circle, thin-dashed circle, thick circle, and eye estimation measurement methods were 0.626, 0.681, 0.681, and 0.462, respectively. There was poor agreement for eye estimation measurements, whereas there was moderate agreement for measurements made using the thin full circle, thin-dashed circle, and thick circle methods.

## Discussion

The highest ICC value among the four methods was obtained in measurements made using the thin full circle. Three key challenges were encountered in measuring the alpha angle: selecting the circle that best fits the femoral head, identifying the circle’s centre, and determining point that overflows the circle. These challenges varied depending on the method used. Challenges in measuring the thin-dashed circle include identifying the best-fitting circle and point that overflows the circle due to gaps in the circle. Challenges in measuring with the thick full circle are determining the point that overflows the circle, as the thick line obscures portions of the excess bone, making assessment difficult. For the eye estimation method, accurately determining both the centre of the circle and the circle that best fit the head proved particularly challenging. We believe these methodological challenges played a role in the differences in ICC values, and that the thin full circle is the most effective approach for addressing these issues and providing reliable measurements.

Recent studies are beginning to provide cut-off values for the alpha angle. Gursan *et al*. in 2023 reported that a postoperative 45° Dunn view alpha angle of ≤48.3° was associated with a higher frequency of reaching the PASS value and threshold of ≤48.3°, and had a sensitivity of 0.75 and specificity of 0.69 to predict positivity [16]. Moreover, in 2022, Monahan *et al*. reported a cut-off value of postoperative plain radiography alpha angle of ≤46° associated with a significantly higher rate of returning to sports [17]. In addition to the postoperative alpha angles, Tang *et al*. in 2022 reported preoperative alpha angle on AP radiography of 70° and Launstein alpha angle of 57° for the cut-off values to predict advanced acetabular rim chondral damage [18]. It is important to note that both lower and higher alpha angle values carry clinical significance. Higher alpha angle values are strongly associated with preoperative cartilage damage severity, indicating their importance in predicting the extent of joint damage prior to intervention. Conversely, lower alpha angle values are closely linked to postoperative functional outcomes, such as return to sports and improved patient-reported functional scores. These findings highlight the necessity for precise and standardized measurement methods across the entire spectrum of alpha angle values to enhance both preoperative assessment and postoperative evaluation, particularly for maintaining reliability in research settings. Therefore, considering recently published studies, which have begun to delineate cut-off values for the alpha angle, even a 2–3° degree alpha angle difference is significant in predicting outcomes such as return to sports, functional scores, and chondral damage.

Interobserver reliability for the eye estimation method was moderate and had the lowest intraobserver reliability among all methods (ICC values were 0.588 and 0.462, respectively). Similarly, Nouh et al. concluded that the eye estimation method for measuring had a limited ability to subjectively assess the alpha angle. Therefore, this method of measurement is not reliable for the determination of alpha angles. Despite this, the higher ICC values for the eye estimation method compared to the thin-dashed circle and thick full circle measurement methods show us that there are significant difficulties in terms of reliability [19].

The interobserver reliability of the alpha angle has been rated as poor to moderate in most studies [5–7]. This is because depending on the measurer the technique can give very different results. The importance of standardizing the alpha angle measurement cannot be ignored. Looking at the research published in recent years, studies on Magnetic Resonance Imaging (MRI) and CT-assisted alpha angle evaluation and FAIS diagnosis and investigation of cam deformities have become more popular than studies using plain radiography. However, these assessment methods, which are projected to be very important in the coming years, are unfortunately not as accessible and affordable as simple X-rays in many countries today [20]. Nepple *et al*. reported that the interobserver reliability of the alpha angle in CT-assisted measurement is low (ICC, 0.43, poor agreement) [7]. Also, in the pilot study by Ewertowski *et al*., in which the results of automatic measurement of alpha angle in 3D MR imaging were published, intraobserver reproducibility was good or excellent, while interobserver reproducibility was moderate (intraobserver first measurer 0.77, second measurer 0.93) (interobserver first measurer ICC = 0.45 second measurer ICC = 0.56 moderate) [6]. Advanced techniques and automatic measurement methods such as CT and MRI will certainly provide much more accurate and valid results in the future. However, CT and especially MRI are methods that are difficult to access and are expensive. It will take some time to eliminate these disadvantages and make them as fast and accessible as plain radiographs in daily practice. This study, which we conducted with the aim of standardising the measurements on plain radiographs and increasing their reliability and repeatability, is the first study to investigate the interobserver reliability of circle types. We believe that the reason for the moderate intraobserver reliability is that the measurements were performed with a manual goniometer.

There were some limitations in our study. One of these was that there were two raters, and another limitation was that the intraobserver reliability was assessed through only one rater. Even though the alpha angle is an objective result, the measurement process can give different results depending on the rater. We also see this in many publications with poor or moderate results for interobserver reliability. Another limitation of this study is that the measurements were taken using a manual goniometer, rather than the digital systems that are commonly employed by modern hip arthroscopists for evaluating alpha and LCEA angles. We believe that higher ICC values will be achieved in further studies, if these limitations are removed. In addition, this study is retrospective, with its patient group dating back nearly 10 years, which represents another limitation to consider.

## Conclusion

The results of the study in which we measured digital images with printed transparent goniometers showed that the thin full circle had the highest interobserver reliability and the highest mean alpha angle values compared to the thin dashed, full thick circles, and eye estimation.

## Data Availability

The data underlying this article will be shared on reasonable request to the corresponding author.
